# Evaluation of *Sargassum cristaefolium*, *Sargassum crassifolium*, and *Enhalus acoroides* as feed additives for improving rumen fermentation and mitigating enteric methane emissions *in vitro*

**DOI:** 10.14202/vetworld.2026.1665-1680

**Published:** 2026-04-25

**Authors:** Nurzainah Ginting, Roni Pazla, Rahmat Hidayat, Asmuddin Natsir, Adanan Purba, Galih Ari Wirawan Siregar

**Affiliations:** 1 Animal Science Study Program, Faculty of Agriculture, Universitas Sumatera Utara, Medan 20155, Indonesia; 2 Department of Animal Nutrition and Feed Technology, Faculty of Animal Husbandry, Universitas Andalas, Padang 25175, Indonesia; 3 Department of Animal Nutrition and Feed Technology, Faculty of Animal Husbandry, Universitas Padjadjaran, Bandung, Indonesia; 4 Animal Nutrition Department, Faculty of Animal Science, Hasanuddin University, Makassar, Indonesia; 5 The team of the center of excellence for science, technology and science of higher education institutions to control greenhouse gas emissions in 2025 of Universitas Sumatera Utara, Medan 20155, Indonesia

**Keywords:** enteric methane mitigation, *in vitro* fermentation, marine biomass, methane emissions, ruminant nutrition, *Sargassum crassifolium*, *Sargassum cristaefolium*, *Enhalus acoroides*

## Abstract

**Background and Aim:**

Enteric methane emissions from ruminants contribute substantially to greenhouse gas accumulation and energy loss in livestock systems. In maritime regions such as Indonesia, macroalgae and seagrass represent abundant but underutilized bioresources with potential antimethanogenic properties. This study evaluated the effectiveness of *Sargassum cristaefolium*, *Sargassum crassifolium*, and *Enhalus acoroides* as feed additives for improving rumen fermentation characteristics and mitigating methane production *in vitro*.

**Materials and Methods:**

A completely randomized factorial design was employed using three marine plant species and four inclusion levels (0%, 5%, 10%, and 15%) with six replicates. Parameters assessed included dry matter degradation (DMD), organic matter degradation (OMD), pH, ammonia nitrogen (NH_3_), volatile fatty acids (VFA), gas production, methane (CH_4_), and microbial protein synthesis. Proximate, Van Soest, and phytochemical analyses were performed to determine nutritional composition and bioactive compounds. Data were analyzed using analysis of variance followed by Duncan’s multiple range test.

**Results:**

All three species exhibited favorable nutritional profiles and contained bioactive compounds, including tannins, flavonoids, and saponins. Supplementation at 10% significantly enhanced DMD, OMD, NH_3_, VFA, gas production, and microbial protein synthesis (p < 0.05), with *S. cristaefolium* demonstrating the most pronounced effects. Rumen pH remained within the optimal physiological range (6.60–7.01) across treatments. Methane production decreased significantly at 10% inclusion, with reductions of 39.86%, 29.30%, and 23.92% for *S. cristaefolium*, *S. crassifolium*, and *E. acoroides*, respectively. Although 15% inclusion yielded greater methane suppression, it adversely affected fermentation efficiency and digestibility parameters. Projections indicated that adopting 10% supplementation could reduce methane emissions in North Sumatra Province from 5,286,097,238 kg to 3,645,821,265 kg by 2050.

**Conclusion:**

Supplementation with *S. cristaefolium*, *S. crassifolium*, and *E. acoroides* at 10% optimizes rumen fermentation while effectively mitigating methane emissions. These findings highlight the potential of marine biomass as a sustainable feed additive for improving livestock productivity and environmental performance. Further in vivo studies are warranted to validate long-term efficacy and practical applicability under field conditions.

## INTRODUCTION

A projected increase in the cattle and buffalo population in North Sumatra Province, Indonesia, to 11,123,942 heads by 2050 would substantially increase methane emissions from the livestock sector [1]. This population is estimated to produce 111,239,420 kg of manure, equivalent to 80,092,382,400 L of methane (CH_4_). Such a large volume of CH_4_ would contribute markedly to climate change if no effective mitigation measures are implemented. One promising approach is the use of feed additives containing methane inhibitors [2–4]. The development of such mitigation strategies is also in line with Indonesia’s commitment to reducing greenhouse gas emissions under international climate agreements [5, 6].

Indonesia has strong potential to utilize methane inhibitors derived from seaweed and seagrass because of its extensive marine biodiversity. Its waters contain 325 identified macroalgal species, comprising 103 Chlorophyceae (green algae), 167 Rhodophyceae (red algae), and 55 Phaeophyceae (brown algae), while 14 of the 60 seagrass species reported worldwide are also found in Indonesia [7, 8]. Despite this richness, seaweed is still often regarded as coastal waste that disturbs beach aesthetics and requires costly removal. Large accumulations of fresh *Sargassum* on coastal areas have been reported to reach around 10,000 tons per day, causing serious disruption to the tourism sector [9]. In addition, brown seaweeds are highly productive, with biomass production exceeding that of many other seaweed groups and reaching 3,000 g cm^-2^ year^-1^ [10].

Among marine plants, brown seaweeds such as *Sargassum* spp. are of particular interest because they contain bioactive compounds, including phlorotannins, that can suppress methane formation in the rumen [11]. Previous *in vitro* studies have shown that seaweed supplementation can reduce CH_4_ production in cattle rumen fermentation by up to 98.9% while increasing the molar proportion of propionate after 72 h of incubation [12]. Seaweed supplementation has also been shown to enhance volatile fatty acid (VFA) production and support fermentative microbial populations involved in microbial protein synthesis [13]. These effects may improve livestock productivity by increasing the efficiency of ruminal energy utilization, decreasing energy loss as CH_4_, and redirecting more energy toward milk and meat production [14, 15].

Seaweed also contains phytochemicals such as tannins and flavonoids, which can improve nitrogen utilization by decreasing protein degradation in the rumen and increasing protein bypass to the small intestine, thereby enhancing ruminant performance [16, 17]. In addition, the defaunating activity of tannins and saponins can reduce protozoal populations, increase microbial protein biomass, and enhance amino acid absorption in the gut [18, 19].

Despite growing evidence supporting the antimethanogenic potential of macroalgae, several critical gaps remain in the application of marine biomass as functional feed additives for ruminants. Most previous studies have primarily focused on a limited number of seaweed species under controlled experimental conditions, with less attention given to locally abundant and underutilized marine resources such as *Sargassum cristaefolium*, *Sargassum crassifolium*, and *Enhalus acoroides*. In addition, although reductions in CH_4_ emissions have been widely reported, the interactive effects of different inclusion levels on rumen fermentation characteristics, digestibility, and microbial protein synthesis remain insufficiently characterized.

Furthermore, the role of phytochemical compounds, including tannins, flavonoids, and saponins, in modulating rumen microbial ecology and fermentation efficiency has not been comprehensively integrated with nutritional evaluations. Many studies have emphasized methane mitigation alone, without simultaneously assessing key productivity-related parameters such as dry matter degradation (DMD), organic matter degradation (OMD), VFA, and ammonia nitrogen (NH_3_). Another important limitation is the lack of region-specific projections that link *in vitro* findings to large-scale methane emission scenarios, particularly in rapidly developing livestock systems such as those in Indonesia. Therefore, a comprehensive evaluation integrating phytochemical profiling, fermentation characteristics, and the emission-reduction potential of locally available marine biomass remains lacking.

The present study aimed to evaluate the potential of *S. cristaefolium*, *S. crassifolium*, and *E. acoroides* as feed additives to improve rumen fermentation efficiency and mitigate enteric CH_4_ production *in vitro*. Specifically, this study investigated the effects of different inclusion levels (0%, 5%, 10%, and 15%) of these marine plants on key fermentation parameters, including DMD, OMD, pH, NH_3_, VFA, gas production, CH_4_ emission, and microbial protein synthesis. In addition, phytochemical analyses were conducted to identify bioactive compounds associated with antimethanogenic activity.

This study also aimed to identify the optimal inclusion level that balances methane mitigation with improved fermentation efficiency and nutrient utilization. Furthermore, the findings were extended to estimate the potential reduction of methane emissions from cattle and buffalo populations in North Sumatra Province under future production scenarios. By integrating nutritional, microbiological, and environmental perspectives, this study seeks to provide a scientific basis for the utilization of locally available marine biomass as sustainable feed additives in ruminant production systems.

## MATERIALS AND METHODS

### Ethical approval

This study did not involve any live experimental animals. All procedures were conducted using rumen fluid obtained from healthy buffalo at a local slaughterhouse (official municipal abattoir) immediately after routine slaughter. The animals were not slaughtered for research purposes, and no additional handling, restraint, or invasive procedures were performed beyond normal abattoir operations. The use of rumen fluid as a by-product of slaughter is exempt from ethical review under the guidelines of the Animal Ethics Committee of Universitas Sumatera Utara and conforms to the Indonesian Ministry of Agriculture Regulation No. 14/2019 on the Welfare of Livestock and Animal Handling During Slaughter.

### Study period and location

This research was conducted between May and October 2024. Samples were taken from Pane Island and analysis was conducted at the Animal Science Study Program, Faculty of Agriculture, Universitas Sumatera Utara, Medan, Indonesia.

### Research materials

**Figure 1:**
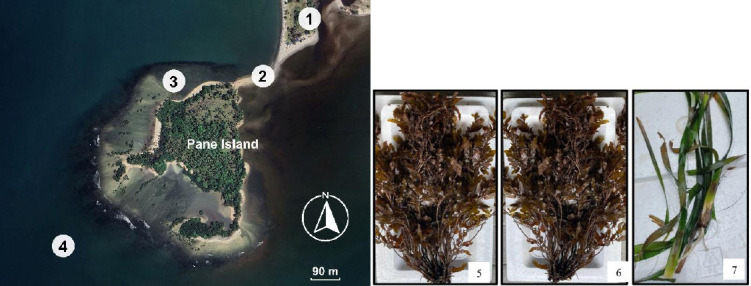
Location of sampling sites. 1. Mainland island, namely Sumatera Island. 2. Shallow land can be traveled at low tide, 10 am to 1 pm. 3. Sampling location, coordinates 1°56’22.78”N and 98°29’54.07”E. 4. Indian Ocean. 5. *Sargassum cristaefolium*. 6. *Sargassum crassifolium*. 7. *Enhalus acoroides*

Seaweed/seagrass were collected from a location with coordinates 1°56’22.78”N and 98°29’54.07”E at Pane Island (Figure 1). The collection was conducted during low tide in the dry season (May-August 2024). Along the coast of Pane Island, marine plants were found stranded on the beach and became waste. However, sampling was conducted in waters with a depth of about 1 m by hand. Seaweeds were found attached to corals, whereas seagrass grew in parts of the water where there were not many corals. Identification of seaweed/seagrass was conducted based on a morphological study by referring to the book “FAO Species Identification Guide for Fishery Purposes: The Living Marine Resources of the Western Central Pacific” [20]. The most common macroalgae at Pane Island were *S. cristaefolium*, *S. crassifolium*, and one seagrass species, *E. acoroides*. *S. cristaefolium* and *S. crassifolium* have similar morphology, except for their leaves. *S. cristaefolium* has comb-like leaves, while *S. crassifolium* has thicker, slightly rounded leaves.

This study focused on two types of brown algae and one seagrass, considering that one of the requirements for an ingredient to be used as animal feed is its availability and nutritional value. Meanwhile, red algae are also present; however, Roskam *et al*. [21] reported that they can cause negative health effects. Red algae contain bromoform, which is carcinogenic.

The seaweed samples were sorted and washed with fresh water to remove embedded dirt, kept in a cool box, and brought to the Nutrition Laboratory, Animal Science Study Program, Faculty of Agriculture, Universitas Sumatera Utara. The samples were then washed again in running water for 5 h to remove salt. Previous research [22] conducted soaking for 3 h in running water. However, in this study, soaking for 5 h gave better results in removing the inherent salt. Furthermore, the samples were oven-dried at 40°C until completely dry, then pulverized in a grinder to a 30-mesh size. The pulverized seaweed and seagrass, together with other ingredients, were formulated into a ration formula for cattle/buffalo. The other ingredients were palm kernel meal, soybean groats, rice bran, fermented corn straw, cassava, coffee grounds, and salt.

### Research methods

This study used a Complete Random Factorial Design with two types of seaweed and one type of seagrass, with four levels of use and six repetitions. The composition of the study treatments is shown in Table 1.

**Table 1:** Composition of the study treatments.

Ingredients	0	5	10	15
*Sargassum cristaefolium*	ScL0	ScL5	ScL10	ScL15
*Sargassum crassifolium*	ScrL0	ScrL5	ScrL10	ScrL15
*Enhalus acoroides*	EaL0	EaL5	EaL10	EaL15

Sc = *Sargassum cristaefolium*, Scr = *Sargassum crassifolium*, Ea = *Enhalus acoroides*, L = level

### Phytochemical analysis

Phytochemical analysis was conducted at the Phytochemical Laboratory, Faculty of Pharmacy, Universitas Sumatera Utara. Macroalgae contain many polyphenolic secondary metabolites, including tannins. Tannins derived from seaweed are called phlorotannins [23]. In this study, phytochemical analysis focused on metabolites correlated with reduced methane formation in the rumen, namely tannins/phlorotannins, flavonoids, and saponins.

**Analysis of total tannin content in *S. cristaefolium*, *S. crassifolium*, and *E. acoroides:*** Before measuring total tannin levels, the samples were macerated. A total of 250 g of simplicia from *S. cristaefolium*, *S. crassifolium*, and *E. acoroides* were dissolved using ethanol for processing by maceration (the principle of maceration is the diffusion of a solvent into plant cells containing active compounds). Total tannin levels in these three simplicia samples were determined using Folin-Ciocalteu reagent and standard tannic acid [24]. Folin-Ciocalteu reagent was used because tannins react with Folin to form a blue solution whose absorbance can be measured. The choice of tannic acid as the standard curve was based on the fact that tannic acid belongs to the class of hydrolyzable tannins and can be used as a reference in measuring total tannin levels. The optimal maximum wavelength of 724.5 nm was determined from the tannic acid standard curve. The sample solution was then measured using a spectrophotometer at 724.5 nm, the wavelength corresponding to the maximum absorbance on the standard curve.

% tannins was calculated using the formula: % tannin = (A × B × C)/D × 100%

Where, A: Concentration from the curve (mg/L), B: Dilution factor, C: Sample volume (L), D: Sample mass (mg)

**Analysis of total flavonoid content in *S. cristaefolium*, *S. crassifolium*, and *E. acoroides:*** Flavonoid identification was performed qualitatively using microscopic and microchemical methods [25]. Methanol extracts from the three samples were placed in test tubes and then identified with and without reagents. Identification without reagents was performed by adding 5 drops of water to the test tube, while identification with reagents involved adding several reagents: 25% NH_3_, 40% NaOH, and 5% AlCl_3_ (5 drops each). The reagents used in this study, at concentrations of 25% NH_3_, 40% NaOH, and 5% AlCl_3_, were based on the premise that these reagents can produce color when flavonoids are present in seagrass leaf extract. The quantification of flavonoid levels of the three samples was carried out using a spectrophotometric method with quercetin as a standard. The standard curve was obtained by measuring the absorbance of the standard quercetin solution at concentrations of 5, 10, 15, 20, and 25 ppm. The maximum wavelength determined for absorbance was 431 nm.

% flavonoid was calculated using the formula: % flavonoid = (KTF (mg/g))/1,000 × 100%

KTF = Total flavonoid content (mg QE/g extract).

**Analysis of total saponin content in *S. cristaefolium*, *S. crassifolium*, and *E. acoroides:*** Saponin content testing (quantitative test) was conducted by the gravimetric method. Saponin content testing was carried out with modifications [26]: 500 mg of extract from each of the three samples was placed in test tubes. Add 10 mL of hot water, cool, and then shake vigorously for 10 s until a firm foam forms. Then add 1 drop of HCl along the side of the test tube. If the foam persists after adding 1 drop of HCl, the sample contains saponins. A sample contains saponins if a stable foam of 1-3 cm in height forms for 30 s.

1.25 g of the extract was refluxed with 50 mL of petroleum ether at 60°C–80°C for 30 min. After cooling, the petroleum ether solution was discarded, and 50 mL of ethyl acetate was added to the residue.

The solution was then extracted by transferring it to a separating funnel and separating the ethyl acetate solution. The residue was dissolved in 50 mL of *n*-butanol three times. The *n*-butanol solutions were mixed and evaporated in a water bath. The remaining precipitate was dissolved in 10 mL of methanol, then added dropwise to 50 mL of ether while stirring.

The precipitate formed was poured onto filter paper of known weight, dried, and weighed to a final weight. The saponin content was calculated from the difference in weight of the filter paper before and after filtration.

% saponin was calculated using the formula: % saponin = (X2 - X1)/A × 100%

Where, X1: Filter paper weight (g), X2: Weight of filter paper + saponin sediment (g), A: Weight of extract sample (g).

The results of phytochemical analysis in this study are presented in Table 2.

**Table 2:** Phytochemical compounds of macroalgae from Pane Island: *Sargassum cristaefolium*, *Sargassum crassifolium*, and Enhalus acoroides.

Phytochemical analysis result	*Sargassum cristaefolium*	*Sargassum crassifolium*	*Enhalus acoroides*
Tannins	0.82	0.70	1.1
Flavonoids	0.32	0.30	0.13
Saponins	0.95	0.91	2.1

### *In vitro* analysis

*In vitro* analyses were conducted in the Ruminant Nutrition Laboratory of the Animal Science Study Program at Universitas Sumatera Utara and the Integrated Laboratory at Universitas Sumatera Utara.

The *in vitro* process was conducted using two methods: the Tilley and Terry method to determine fermentability, digestibility, and total microbial counts [27], and a simpler modified technique by Fieves *et al. [28]*, using a gas syringe to determine gas production. The Tilley and Terry method was carried out as follows: 0.75 g of complete feed from each treatment was placed in a 100 mL infusion bottle, and then 25 mL of rumen fluid and 50 mL of McDougall’s solution were added. The bottle was tightly closed to ensure airtightness and incubated to mimic the atmosphere inside a buffalo rumen. In this study, rumen fluid was collected from healthy buffalo at a local slaughterhouse within 15 min of slaughter. The slaughterhouse is located 5 km from the campus laboratory. Rumen fluid was stored in pre-warmed thermos flasks, and *in vitro* incubation began immediately, approximately 20 min later, to ensure microbial viability. The buffalo were fed fresh local corn stalks, cassava peels, and rice bran twice daily. The rumen fluid pH measured at the time of sampling was 6.85.

### Dry matter and organic matter digestion coefficient analysis

The laboratory research method used for *in vitro* digestibility was the Tilley and Terry method [27]. This method was implemented as follows: The water bath was first filled with water up to one-third of the height of the test tube. Then, the water bath was turned on and the temperature was set to 39°C. The air-dried sample with known dry matter content was weighed to 0.50 g and then placed into a 100 mL test tube. Next, a mixture of rumen fluid and artificial saliva (10:40 mL) was poured into the test tube while it was shaken. Another test tube was filled with a mixture of rumen fluid and artificial saliva (10:40 mL) as a blank, then the test tube was closed, injected with CO_2_ gas, placed in a water bath rack, and tightly sealed. The test tube was then shaken slowly every 8 h. After 48 h of incubation, acid pepsin solution was added (0.2% pepsin, 2 mL, and 0.1 N HCl, 6 mL). The incubation process was then continued for 84 h. After that, the contents of the test tube were filtered into a crucible and rinsed with warm water (75°C) until the test tube was free of sample residue. The crucible and sample were then placed in an oven at 105°C until a constant weight was obtained (±2 days). Next, the crucible and dried sample, as well as the blank, were weighed to determine the residual weight of the sample and blank. The DMD coefficients were calculated using the equation:

DMD = (DMi - (DMr - DMb))/DMi × 100%

Where, DMD: Dry matter digestibility, DMi: Dry matter initial, DMr: Dry matter residue, DMb: Dry matter blank.

To obtain the digestibility value of organic feed material, the dry material residue is burned in a crucible in a furnace at 500°C–600°C for 2–3 h, or until the color is grayish white. The resulting ash is then weighed; the difference between the dry material and the ash is the organic material. Then, the sample is weighed to determine the weight of the sample residue (organic material) and the weight of the blank residue (organic material). The digestibility of the sample organic material is calculated using the formula:

OMD = (OMi - (OMr - OMb))/OMi × 100%

Where, OMD: Organic matter digestibility, OMi: Organic matter initial, OMr: Organic matter residue, OMb: Organic matter blank

### Total NH_3_ level analysis

Total NH_3_ levels were analyzed using the Conway microdiffusion method. The Conway cup (Iwaki glass, Gede Bage, Jawa Barat, Indonesia) was first smeared with vaseline on the lips. A total of 1 mL of supernatant was placed on one side of the cup partition, and 1 mL of saturated Na_2_CO_3_ solution was placed on the other side. The cup was tilted toward the partition so that the two solutions did not mix. In the center of the cup, 1 mL of boric acid was placed. The Conway cup, with its lip smeared with vaseline, was then tightly closed to make it airtight. The saturated Na_2_CO_3_ solution was mixed with the supernatant by shaking and tilting the cup. The cup was then left at room temperature (28°C) for 24 h. After that, the cup lid was opened, and the boric acid was titrated with 0.005 N H_2_SO_4_ until the color changed from blue to reddish. The NH_3_ level was calculated using the formula:

N-NH_3_ (mM) = mL titration H_2_SO_4_ × N H_2_SO_4_ × 1,000

Where, N-NH_3_ = Concentration of N-ammonia (mM), N H_2_SO_4_ = Normality of H_2_SO_4_ solution

### Total VFA analysis

The total concentration of VFA was determined using the “steam distillation” method (General Laboratory Procedure, 1996). A total of 5 mL of supernatant was collected and placed in a distillation tube. Then, 1 mL of 15% H_2_SO_4_ was added, and the tube was immediately closed with its lid to ensure airtightness and connected to a cooling flask (Liebig, Fisher Scientific, Waltham, Massachusetts, USA). Immediately after adding 15% H_2_SO_4_ to the supernatant, the tube was inserted into a distillation flask containing boiling water (heated during distillation). The hot water vapor that displaces the VFA condenses in the cooler. The water formed was collected in an Erlenmeyer flask containing 5 mL of 0.5 N NaOH solution until it reached about 250 mL. Two drops of phenolphthalein (PP) indicator were added to the collected distillate, and the distillate was titrated with 0.5 N HCl until the color changed from pink to colorless.

### The procedure can be seen in the following formula:

Total VFA = (b – s) × N HCl × 1,000/5 mM

Where, s = Volume of sample titrant, b = Volume of blank titrant, N = Normality of HCl solution

### CH_4_ reduction analysis

To obtain CH_4_ reduction, total gas and methane (CH_4_) production must be measured using a simpler modified technique of Fieves *et al. [28]*, namely, using a gas syringe. The rumen fluid for fermentation used in this study was derived from the rumen of a local slaughterhouse within 15 min of slaughter. The rumen fluid was placed in a preheated thermos flask. *In vitro* analysis was performed immediately upon arrival at the campus laboratory, located 5 km from the slaughterhouse, to maintain microbial viability.

The Tilley and Terry method was conducted as follows: 0.75 g of complete feed from each treatment was placed in a 100 mL infusion bottle, and then 25 mL of rumen fluid and 50 mL of McDougall’s solution were added. The bottle was sealed to make it airtight and conditioned to resemble the atmosphere in cattle’s rumen. Gas production was measured at 2, 4, 6, 8, 10, 12, and 24 h.

The volume of produced gas was converted to a per-gram ratio of 1 g of digested organic matter. Methane was separated from other gases by passing it through a 10 M NaOH solution, which absorbs CO_2_ and other acid gases. The remaining methane gas was recorded as mL/g digested organic matter.

Methane gas production for each treatment, including the control, was obtained. To obtain the methane reduction rate, methane production for each treatment was subtracted from that of the control. To obtain the percentage, the reduction result is multiplied by 100%.

### Statistical analysis

The data obtained from the study were statistically processed using variance analysis. All collected data were processed and analyzed for variability using two-way analysis of variance with SPSS software version 25.0, followed by Duncan’s multiple range test [29].

## RESULTS AND DISCUSSION

### Nutrient content of *S. cristaefolium, S. crassifolium*, and *E. acoroides*

The nutrient content of all three algae was favorable, with crude protein levels ranging from 7.12% to 9.36% (Table 3). For use as animal feed, this protein content is comparable to that of cultivated grasses such as *Pennisetum purpureum*, *Setaria sphacelata*, and *Brachiaria mutica*, which are commonly used in livestock fattening in Indonesia. Total digestible nutrients were also satisfactory, indicating relatively good digestibility because these materials contain more readily digestible fiber. The fat content of all three algae was low, which is typical of algae. The ash content was relatively high, reflecting their mineral content.

**Table 3:** Nutrient content of *Sargassum cristaefolium, Sargassum* *crassifolium*, and *Enhalus acoroides.*

Nutrients (% of Dry matter basis)	Seaweed		Seagrass

	*S. cristaefolium*	*S. crassifolium*	*E. acoroides*
Dry matter	81.34	80.56	82.21
Crude protein	8.52	7.12	9.36
Neutral detergent fiber	18.15	15.35	19.12
Acid detergent fiber	23.23	20.12	24.34
Ether extract	1.90	1.85	2.10
Ash	30.23	29.41	31.52

### Phytochemical content of *S. cristaefolium, S. crassifolium*, and *E. acoroides*

Algae contain phytochemical compounds that are beneficial as antioxidants, enzyme stimulants, anti-inflammatory agents, and bacterial growth inhibitors (Table 4). Therefore, these plants should be utilized as widely as possible for consumption, including as livestock feed. Algae have not been utilized by residents on the west coast of Sumatera Utara Province for human consumption, let alone for livestock feed. Pane Island is located on the west coast of Sumatera Utara Province, in Central Tapanuli Regency, which has the largest buffalo population in Sumatera Utara Province.

**Table 4:** Feedstuff composition and chemical components of each complete feed containing different levels of macroalgae.

Percentage	Control	*Sargassum cristaefolium*			*Sargassum crassifolium*			*Enhalus acoroides*		
Macroalgae	0	5	10	15	5	10	15	5	10	15
Palm kernel meal	22	22	22	22	22	22	22	22	20	20
Soybean groats	10	9.5	10	9	10	10	9	10	8	8
Rice bran	13	12	11	9	9	7	6	8	9	7
Corn ash	8	7	7	7	9	9	7	8	9	9
Fermented corn straw	35	33	31	29	33	30	29	35	33	30
Coffee grounds	11	10.5	8	8	11	11	11	11	10	10
Mineral	1	1	1	1	1	1	1	1	1	1
Total	100	100	100	100	100	100	100	100	100	100
Nutrient (%)										
CP	12.02	12.04	12.07	12.48	12.02	12.03	12.27	12.14	12.45	12.60
CF	23.24	23.30	23.41	23.51	23.16	23.28	24.93	25.32	25.60	25.71
EE	4.62	4.53	4.21	4.21	4.42	4.45	4.35	4.62	4.48	4.43
NFE	47.32	47.30	47.35	47.18	47.14	45.31	47.41	47.31	47.42	47.80
TDN	64.0	62.9	62.8	62.42	62.42	62.03	63.02	60.91	60.75	60.71

DM = dry matter, OM = organic matter, CP = crude protein, CF = crude fiber, EE = ether extract, NFE = nitrogen-free extract, TDN = total digestible nutrients

Flavonoids were detected in this study. Flavonoids have previously been reported in *Sargassum* and are known to possess antibacterial and anti-inflammatory activities [30]. *S. crassifolium* contained relatively high flavonoid levels, which may be associated with its antioxidant capacity [31]. *E. acoroides* also contained flavonoids, and previous work has shown that flavonoid content in *Enhalus* increases with plant age [32].

Tannins were detected in *S. cristaefolium*, *S. crassifolium*, and *E. acoroides*. The genus *Sargassum* has been reported to consistently contain tannins [33]. This alga is favored by herbivorous fish, possibly because of the beneficial antioxidant effects of tannins. *E. acoroides* has also been reported to contain tannins that are beneficial for digestive health because they support the growth of beneficial bacteria [34].

Saponins were found in *Sargassum*, which is consistent with the findings of Amrillah [35]. Saponins function as anti-inflammatory, antimicrobial, and antiviral agents, and they also enhance immune function by stimulating T-cell production, acting as antioxidants, and reducing oxidative stress [36]. Therefore, the presence of saponins is important for improving feed quality.

The presence of phytochemicals in these three algae indicates that they have the potential to be used as animal feed additives. The phytochemical content of seaweed is closely associated with its health benefits [37]. Communities living on the southern coast of Aceh consume *Sargassum* sp. and *Enhalus acoroides*, and these plants are also used as livestock feed. In contrast, communities around Pane Island do not yet utilize these algae. In this study, the taste of the three algae was also evaluated. *S. cristaefolium* and *S. crassifolium* tasted like cucumber and were crunchy and slightly sweet, whereas *E. acoroides* tasted slightly bitter. The taste of *E. acoroides* differs because its tannin content is higher than that of *Sargassum*.

Brown algae, including *S. cristaefolium*, *S. crassifolium*, and *E. acoroides*, are commonly used as animal feed in Boholano, Philippines [38]. The benefits of *Sargassum* as feed are related to its chemical constituents, such as carotenoids, fucoxanthin, and polysaccharides, namely alginates, laminarins, fucans, and cellulose. The use of algae as livestock feed also occurs on the remote Scottish island of North Ronaldsay, where sheep routinely consume algae washed ashore, which make up around 80% of their diet [39]. The use of algae for livestock feed in the Philippines, Aceh, and Scotland since ancient times, together with their nutritional and phytochemical contents, demonstrates that algae have strong potential as alternative livestock feed resources. For maritime countries such as Indonesia, livestock feeding strategies need to use algae rather than relying solely on agricultural raw materials, especially as livestock demand increases with population growth.

### DMD

The increase in DMD was mainly associated with increasing algae concentration. In this study, 10% algae tended to yield higher DMD, and *S. cristaefolium* showed higher DMD than *S. crassifolium* and *E. acoroides*. There was an interaction between the type of algae and the inclusion level. A previous study found that a ration containing 10% *Sargassum* sp. was better than a ration containing 20% *Sargassum* sp. [40]. DMD in that study was 64.04% and 61.66% at 10% and 20% *Sargassum*, respectively, whereas in this study, it was 57.59% at 10% *Sargassum* and decreased to 50.12% at 15% (Table 5). In the feed formula used by [40], the percentage of wheat bran and millet corn decreased with the addition of *Sargassum*, whereas in this study, fermented corn straw and rice bran decreased as *Sargassum* increased. Another study found that the DMD of a combination ration of *Sargassum* sp. and corn starch was not significantly different from that of a pollard bran ration, and reported no difference in weight gain between the two rations in sheep fattening [41]. Another study found that pelagic *Sargassum*, which contains brown algae at up to 30%, increased DM when included in rations containing *P. purpureum* grass hay [42]. In addition, the presence of algae in rations can trigger the growth of cellulolytic microbes such as *Fibrobacter succinogenes* and *Ruminococcus flavefaciens*, thereby affecting DM digestibility [42]. Differences in dry matter values may be caused by climate, algal age, nutrient supply, and algal species. The DMD of *E. acoroides* also decreased at 15% compared with 10%, from 53.33% to 47.21%. Research on seagrass has shown that seagrass can positively affect DMD and can replace oat hay in alpine goat diets [43].

**Table 5:** Table of F value and p-value.

Parameters	Treatment 1 (*Sargassum* *cristaefolium*, *Sargassum* *crassifolium*, and *Enhalus acoroides*)		Treatment 2 (Level 0%, 5%, 10%, and 15%)		Interaction between Treatment 1 and Treatment 2	

	F value	p-value	F value	p-value	F value	p-value
Dry matter digestibility	47.871	0.000	129.231	0.000	6.095	0.000
Organic matter digestibility	46.913	0.000	109.538	0.000	6.901	0.000
pH	1.841	0.168	1.681	0.181	0.498	0.807
NH₃ (mM)	10.069	0.000	28.015	0.000	1.378	0.238
VFA (mM)	1181.151	0.000	1922.958	0.000	171.960	0.000
Acetic acid (mM)	33.971	0.000	334.353	0.000	9.182	0.000
Propionic acid (mM)	1.287	0.284	290.104	0.000	0.287	0.941
Gas production (mL)	492.109	0.000	379.115	0.000	69.402	0.000
CH₄ (ppm)	65992783.67	0.000	1062198376	0.000	10076715.10	0.000
CH₄ % reduction compared with control	1252.032	0.000	20144.063	0.000	191.078	0.000
Microbial protein synthesis (mg/100 mL)	1231.554	0.000	13638.733	0.000	229.837	0.000
Bacteria (10⁹)	30.663	0.000	415.332	0.000	4.233	0.001
Protozoa (10⁵)	70.892	0.000	315.237	0.000	16.548	0.000

### OMD

The addition of *Sargassum* to feed has been reported to improve OMD up to 30% inclusion [42]. This is because *Sargassum* contains non-fibrous carbohydrates and minerals that provide energy for rumen microbes. *Sargassum* contains glucose, galactose, and mannose, which support the growth of rumen microbes and the host [44]. *In vitro* tests indicate that rumen microbes need time to adapt to new substrates. This is evidenced by the finding that rumen microbes required 72 h of adaptation, after which rapid growth ensued, resulting in similar digestion rates across treatments with 10%, 20%, or 30% *Sargassum*. This information provides a practical solution for farms that attempt to feed seaweed to livestock, which are often said to dislike it. Feeding, therefore, needs to continue until the rumen microbes adapt, thereby accelerating digestion and feed intake. In this study, 10% *S. cristaefolium* had an OMD value of 67.41%, whereas in the study by [40], OMD was 51%. The better OMD in this study may be related to preparation quality, as the algae were washed in running water for 5 h, whereas Budi washed them only 3 times. High salt concentration can inhibit rumen microbial activity. In addition, the algae in this study were collected from clean waters without nearby human settlements.

This study showed an increase in OMD with 10% supplementation in the ration. There was an interaction between the type of algae and the inclusion level. *E. acoroides* has been reported to contain the highest soluble fiber among several seagrasses, namely *E. acoroides* (62.4%), followed by *C. rotundata* (54.1%), *T. hemprichii* (50.9%), and *S. isoetifolium* (43.3%) [45]. Soluble fiber is beneficial for livestock because it prolongs the digestive process in the rumen, allowing more nutrients to be digested with the help of rumen microbes.

### pH of rumen fluid

The pH of rumen fluid varied with the type of seaweed or seagrass and its concentration. There was no significant effect on pH, with values ranging from 6.60 to 7.01 (Table 6). A rumen pH range of approximately 6.9 to 7.3 is categorized as normal because it can support rumen microbial growth [46, 47]. Rumen pH is influenced by feed type. In this study, rumen pH may have been influenced by one of the feed ingredients, namely fermented corn waste, which had a pH of 5.15 [48]. The pH values in this study were slightly lower than those reported in another study, which ranged from 6.67 to 7.44 using elephant grass as the forage source [49].

**Table 6:** The effect of *Sargassum cristaefolium*, *Sargassum crassifolium*, and *Enhalus acoroides* on digestibility and fermentation process.

Parameters	C	*Sargassum cristaefolium*			*Sargassum crassifolium*			*Enhalus acoroides*		
Macroalgae	0	5	10	15	5	10	15	5	10	15
Dry matter digestibility	51.31^b^ ± 1.139	55.12^de^ ± 1.419	57.59^f^ ± 1.298	50.12^b^ ± 0.612	54.76^d^ ± 0.893	56.11^e^ ± 0.938	50.09^b^ ± 0.721	51.13^b^ ± 1.052	53.33^c^ ± 1.163	47.21^a^ ± 0.901
Organic matter digestibility	64.12^c^ ± 1.088	65.38^cd^ ± 0.941	67.41^d^ ± 0.962	60.21^b^ ± 0.741	64.23^c^ ± 1.466	66.76^d^ ± 1.505	60.56^b^ ± 1.762	60.71^b^ ± 1.26	63.89^c^ ± 1.298	55.43^a^ ± 0.679
pH	6.81 ± 0.1	6.71 ± 0.179	6.60 ± 0.258	6.71 ± 0.171	6.76 ± 0.192	6.70 ± 0.141	6.73 ± 0.166	6.82 ± 0.138	6.79 ± 0.084	6.75 ± 0.1501
NH₃ (mM)	11.10 ± 0.211	12.31 ± 1.232	13.65 ± 0.754	12.91 ± 0.606	12.21 ± 0.526	13.41 ± 0.692	12.64 ± 0.828	11.24 ± 0.677	12.23 ± 0.815	12.06 ± 0.708
VFA (mM)	75.11ᶦ ± 0.713	70.31^g^ ± 0.771	72.65^h^± 0.388	66.21^f^ ± 0.687	66.23^f^ ± 1.012	60.51^d^ ± 0.473	56.64^b^ ± 0.558	61.34^e^ ± 0.742	58.71^c^ ± 0.497	52.46^a^ ± 0.552
Acetic acid (mM)	30.26^a^ ± 0.689	34.53^c^ ± 0.718	39.12^e^ ± 0.664	33.74^c^ ± 0.708	31.47^b^ ± 0.775	37.90^d^ ± 1.025	33.98^c^ ± 0.696	30.96^ab^ ± 0.884	37.44^d^ ± 0.894	31.60^b^ ± 0.808
Propionic acid (mM)	25.42 ± 1.326	16.41 ± 1.199	18.37 ± 0.789	16.58 ± 0.546	15.82 ± 0.983	17.97 ± 1.407	16.66 ± 1.046	15.65 ± 0.769	17.81 ± 1.157	15.87 ± 0.925
Gas production (mL)	107.21^f^ ± 0.38	109.11^h^± 0.477	111.25ᶦ ± 0.733	103.78^d^ ± 0.502	106.12^e^ ± 1.138	108.15^h^± 0.446	102.31^c^ ± 0.382	101.22^b^ ± 0.751	103.15^d^ ± 0.337	99.52^a^ ± 0.246
CH₄ (ppm)	21,982.78^j^ ± 0.766	17,380.21^g^ ± 0.744	13,219.45^d^ ± 0.799	5,085.71^a^ ± 0.402	17,985.47^h^± 0.7496	15,542.21^e^ ± 0.7494	7,638.20^b^ ± 1.042	19,825.21ᶦ ± 1.159	16,723.21^f^ ± 0.732	9,789.53^c^ ± 0.734
CH₄ % reduction compared with control	-	20.94^d^ ± 1.278	39.86^g^ ± 0.858	76.87^j^ ± 0.999	18.18^c^ ± 0.656	29.30^f^ ± 1.086	65.25ᶦ ± 1.143	9.81^b^ ± 0.743	23.92^e^ ± 0.826	55.47^h^± 0.963
Microbial protein synthesis (mg/100 mL)	252.54ᶦ ± 0.769	218.03^f^ ± 0.615	229.52^h^± 0.411	217.25^f^ ± 0.457	208.72^b^ ± 0.421	219.21^g^ ± 0.811	212.16^e^ ± 0.966	204.45^a^ ± 0.937	209.57^c^ ± 0.656	210.67^d^ ± 0.609
Bacteria (10⁹)	99^f^ ± 2.606	63.2^de^ ± 3.688	67.6^e^ ± 6.449	66.5^e^ ± 4	54.2^ab^ ± 2.607	57.1^bc^ ± 2.828	51.5^a^ ± 1.414	60.5^cd^ ± 7.071	67.1^e^ ± 5.441	61.3^cd^ ± 1.41
Protozoa (10⁵)	86^f^ ± 1.414	75.2^e^ ± 3.506	73.8^f^ ± 4.243	66.5^cd^ ± 3.847	61.2^b^ ± 2.828	60.2^b^ ± 2	59.1^b^ ± 2.82	69.5^d^ ± 2	65.2^c^ ± 3.406	52.3^a^ ± 1.42

A rumen pH within the normal range indicates that the substrate composition does not interfere with rumen microbial activity. The opposite condition may cause extreme pH fluctuations. For example, if pH becomes too low or too high, microbial activity and nutrient digestion are disrupted. If rumen pH falls below 6, proteolysis and deamination are disturbed. If rumen pH exceeds 7.3, ammonia absorption increases [50]. The normal pH recorded in this study for each treatment indicated that adding *Sargassum* and *E. acoroides* at 5%-15% supported rumen microbial degradation of the substrate. Therefore, organic matter was broken down into components that were more readily digested by rumen microbes, so that the production of VFA and NH_3_ did not interfere with rumen pH [51].

### NH_3_ production

In this study, NH_3_ production did not differ significantly among algae levels, and all values remained within the normal NH_3_ production range (Table 6). NH_3_ production was highest at the 10% algae level for *S. cristaefolium*, *S. crassifolium*, and *E. acoroides*, with values of 13.65, 13.41, and 12.23 mM, respectively. Treatment with phlorotannin extract from the brown algae *Ascophyllum nodosum* has been reported to produce NH_3_ at a level of 12.52 [52]. Another study found that treatment with 2%, 5%, and 10% brown algae (*Sargassum mcclurei*) produced NH_3_ levels of 13.29, 13.92, and 14.86 mM/L, respectively, with 10% *S. mcclurei* showing the highest NH_3_ yield [53]. The present study similarly found that 10% *S. cristaefolium*, *S. crassifolium*, and *E. acoroides* produced higher NH_3_. NH_3_ supports microbial protein synthesis in the rumen as a nitrogen source [54]. NH_3_ production that is too low or too high disrupts rumen microbial activity. Excessive NH_3_ production causes nitrogen imbalance and energy loss because surplus NH_3_ must be excreted through urea metabolism [55]. Conversely, too little NH_3_ also disrupts rumen microbial activity and feed efficiency. The normal NH_3_ levels observed in this study likely resulted from feed formulas adjusted to livestock requirements and supplemented with algae levels that did not impair rumen microbial activity but instead supported better nitrogen utilization.

### The effects of *S. cristaefolium, S. crassifolium*, and *E. acoroides* on VFA

In this study, VFA values at 10% *S. cristaefolium* differed from those of all other treatments (Table 6). There was an interaction between algae type and algae percentage. This may be related to the higher concentration of phlorotannins, which suppresses rumen microbial activity. The VFA values of treatments supplemented with *S. cristaefolium*, *S. crassifolium*, and *E. acoroides* at all levels were lower than those of the control. Brown seaweed treatment has been shown to reduce bacterial populations and VFA volume [52]. That reduction in VFA was consistent with changes in rumen microbial populations [52]. The results of this study were similar to those findings.

### The effects of *S. cristaefolium, S. crassifolium*, and *E. acoroides* on gas production

High gas production indicates effective fermentation of organic matter, but excessive gas production can be detrimental because part of that gas is methane, which is environmentally harmful and reduces livestock energy efficiency [14]. In this study, treatments containing 10% *S. cristaefolium*, *S. crassifolium*, and *E. acoroides* showed higher gas production, whereas gas production decreased at the 15% level.

### The effects of *S. cristaefolium, S. crassifolium*, and *E. acoroides* on CH_4_

During enteric fermentation, rumen microbes degrade feed into VFA, a process that also produces CO_2_ and H_2_. These gases then serve as substrates for CH_4_ formation by methanogenic archaea [56]. CH_4_ is harmful to the environment because it contributes to global warming. In addition, this energy could otherwise be used for growth or milk production. Around 2%-12% of energy is lost as gas; therefore, research on modifying conventional feed ingredients in ration formulas is needed to minimize enteric CH_4_ production [54].

Research has been conducted on brown algae such as *Sargassum fusiforme* and *S. falfallum*, which are selected for their abundant production, diverse phytochemical content, and suitability for use as animal feed [57]. That study showed that CH_4_ decreased, as did its proportion relative to total gas, after 12, 24, and 48 h of incubation, even though total gas did not differ significantly [57].

Research on *Sargassum* sp. at concentrations of 0%, 5%, 10%, and 15% found that the higher the *Sargassum* sp. concentration, the lower the total gas and CH_4_ production [49]. That study reported CH_4_ production of 6,096.35 ppm at 15% *Sargassum*. In the present study, 15% *S. cristaefolium* produced 5,085.71 ppm CH_4_. *S. cristaefolium* produced lower CH_4_ than *S. crassifolium* or *E. acoroides* at every algae inclusion level (Table 5).

Many studies have investigated the modification of feed formula ingredients using various types of seaweed to manipulate enteric CH_4_ production. Brown seaweed can reduce CH_4_ because of its content of polyphenolic compounds such as phlorotannins [52-54, 57]. Phlorotannins can reduce methane formation [52, 53]. Saponins in algae can also reduce methane [58]. This is because saponins are toxic to protozoa. Saponins also affect the populations of some bacteria, including methanogenic archaea, by damaging their membrane lipids. Thus, saponins may improve the efficiency of ruminant fermentation by reducing methanogenesis.

Research on red, brown, and green seaweeds found that all red seaweed species tested, including *Asparagopsis armata*, *Bonnemaisonia hamifera*, *Euptilota formisissima*, *Plocamium cirrhosum*, and *Vidalia colensoi*, had potential as anti-methanogenic feed, but only *A. armata* contained bromoform as an anti-methanogenic compound [59]. After 48 h of incubation, all seaweeds reduced CH_4_ production by 6%-10%. In addition, seaweed contains secondary metabolites that also act as anti-methanogenic agents [59]. The brown seaweed used in that study was *Ecklonia radiata*, which also has anti-methanogenic properties and contains polyphenols [59].

In the present study, *E. acoroides* at concentrations of 5%, 10%, and 15% reduced CH_4_ by 9.81%, 23.92%, and 55.42%, respectively. *E. acoroides* leaves contain alkaloids, flavonoids, tannins, and steroids, and their tannin content is very high [60].

Although *S. cristaefolium*, *S. crassifolium*, and *E. acoroides* have potential to be used as animal feed at up to 15%, *in vivo* application in the field requires caution regarding the percentage provided. This is because these algae are not conventional feed ingredients, and rumen microbes need time to adapt to them.

### Microbial protein synthesis

Seaweed percentage showed a significant difference (p < 0.05) in microbial protein synthesis among treatments, with microbial protein production during the 48-h incubation period ranging from 204.45 to 229.52 mg/100 mL. The 10% *S. cristaefolium* treatment showed the highest microbial protein synthesis, at 229.52 mg/100 mL. There was an interaction between algae type and algae percentage. Microbial protein production in this study was higher than that reported in another study using 15% *Sargassum* spp., which reported a value of 199.26 mg/100 mL [49]. This may be related to the feed material used in this study, namely fermented corn straw. Fermented corn straw using rumen fermentation extract from buffalo has been shown to contain enzymes and bacteria similar to those found in buffalo rumen [48]. These enzymes include cellulase and hemicellulase, with bacteria such as *Bacteroides succinogenes* and *Ruminococcus albus*. The use of fermented feed produced with rumen extract in this study appears to support rumen microbial activity, including microbial protein synthesis.

Treatment with 2% *Sargassum fulfellum* has been shown to suppress the growth of cellulolytic bacteria such as *Ruminococcus albus*, *Fibrobacter succinogenes*, and *Ruminococcus flavefaciens*. In addition, *Sargassum* also suppressed the growth of methanogenic archaea [57]. Treatment with 10% *Sargassum mcclurei* significantly suppressed the growth of *Ruminococcus* and *Methanomicrobium* [53]. The effects of algal treatment vary depending on the species, season, collection site, and level of administration [57]. The effects of phlorotannin from brown seaweed on rumen bacteria are species-specific because the abundance of cellulolytic bacteria such as *Fibrobacter succinogenes* may decrease, whereas *R. albus* and *R. flavefaciens* may remain unaffected [52]. *S. bovis*, *S. ruminantium*, and *Butyrivibrio fibriosolvens* have been reported to be the predominant rumen bacteria in sheep consuming seaweed containing phlorotannin [61]. The mechanism by which phlorotannins suppress rumen bacterial growth likely involves the structure and chemical composition of the bacterial cell wall. Changes in rumen microbial populations do not necessarily affect protein synthesis efficiency [62].

In this study, the bacterial population was higher than the protozoal population, with bacteria at 10^9^ and protozoa at 10^5^ (Table 5). *S. cristaefolium* had the highest population at the 10% level compared with the other percentages in both *S. crassifolium* and *E. acoroides*. This correlates with the superior nutritional performance of *S. cristaefolium*, as evidenced by higher dry matter and organic matter digestibility.

This study showed that almost all parameters exhibited interaction effects, as indicated by different superscript letters in the data. However, some parameters, such as pH and NH_3_, did not show interaction, and their data therefore did not carry such letter distinctions. Another indication of interaction is a p-value of less than 0.05, as seen in most values in Table 5, many of which were 0.000.

The business-as-usual projection of methane from enteric fermentation of cattle and buffalo in 2024 and the projection of methane by *S. cristaefolium*, *S. crassifolium*, and *E. acoroides* as additives in 2050 in North Sumatra Province, Indonesia

In this study, two different scenarios were evaluated. These were the business-as-usual scenario for methane emissions in the baseline year, without any mitigation effort, and the estimated emission reductions if mitigation actions were implemented by providing feed additives of *S. cristaefolium*, *S. crassifolium*, and *E. acoroides* (Table 6). The cattle and buffalo population in 2050 was projected based on the average growth in cattle and buffalo populations over the last 10 years in North Sumatra Province.

Beef cattle and buffalo produce methane gas emissions using the following formula:

Methane gas production (kg) = Population × 15 × 48 × 0.66

Where 15 = Each cow in Indonesia produces 10 kg of feces, whereas each buffalo produces 20 kg of feces; the average of both is 15 kg. 48 = Each kg of feces produces 48 liters of methane gas [63] 0.66 = Conversion factor to kg of methane gas [64]

Secondary metabolites in brown algae, such as phlorotannin and saponins, have toxic effects on bacteria, protozoa, and methanogenic archaea. Because of commensal relationships, changes in bacterial and protozoal populations also affect methanogenic archaeal populations, consequently decreasing methane production. The findings of this study suggest that 10% *S. cristaefolium*, *S. crassifolium*, and *E. acoroides* can be proposed to disrupt the process of methanogenesis.

In Table 7 [1, 65], the 2050 projection with the addition of 10% *S. cristaefolium*, *S. crassifolium*, and *E. acoroides* as feed additives indicates that the average enteric methane reduction from the three algae is 31.03%, resulting in enteric methane emissions from cattle and buffalo farming of 360,592,591 kg, whereas without algae feed additives, enteric methane emissions would be 5,286,097,238 kg. Through intensive dissemination, it is assumed that the three algae will be used by cattle and buffalo farmers at a 10% inclusion rate in feed. The search for non-conventional feed ingredients is strongly recommended in Indonesia because of climate change. Areas with large buffalo populations in Indonesia are endemic for hemorrhagic septicemia [66], including coastal areas such as Central Tapanuli Regency, where Pane Island is located. During the dry season, buffalo often lack feed, which causes stress and is usually followed by the emergence of hemorrhagic septicemia disease. Farmers in Central Tapanuli Regency can utilize the three types of algae that grow abundantly on Pane Island. Because Indonesia is an archipelago rich in various types of algae, there is hope for improved ruminant health and growth.

**Table 7:** Scenarios of methane production in 2024 and 2050 in North Sumatera Province.

Scenario	Cattle and buffaloes population	Methane production (kg)
BAU 2024	833,004 heads [65]	39,151,188
2050 Projection (Without *Sargassum cristaefolium*, *Sargassum crassifolium*, and *Enhalus acoroides* as feed additive)	11,123,942 heads [1]	5,286,097,238
2050 Projection (With *S. cristaefolium*, *S. crassifolium*, and *E. acoroides* as feed additive)	11,123,942 heads [1]	3,645,821,265

## CONCLUSION

This study demonstrated that the inclusion of *S. cristaefolium*, *S. crassifolium*, and *E. acoroides* significantly influenced fermentation characteristics, digestibility, and CH_4_ production under *in vitro* conditions. A 10% inclusion level consistently produced optimal outcomes, characterized by higher DMD and OMD, improved microbial protein synthesis, and favorable NH_3_ concentrations. Increasing inclusion levels up to 15% resulted in substantial reductions in CH_4_ production, with *S. cristaefolium* showing the strongest antimethanogenic effect. The presence of phytochemicals, particularly tannins (phlorotannins), flavonoids, and saponins, likely contributed to the modulation of rumen microbial populations and suppression of methanogenesis.

The findings highlight the potential of locally available marine biomass as sustainable feed additives. Inclusion levels of 10%–15% can enhance fermentation efficiency while reducing CH_4_ emissions. This dual benefit supports improved livestock productivity and environmental sustainability. In regions such as North Sumatra, where these algae are abundant yet underutilized, incorporating them into feeding strategies can reduce reliance on conventional feed resources and support climate-smart livestock production. Large-scale applications may significantly reduce CH_4_ emissions from cattle and buffalo systems.

This study integrates phytochemical profiling, nutritional evaluation, and *in vitro* fermentation analysis to provide a comprehensive assessment of algae as feed additives. The use of locally sourced species increases practical applicability. The factorial design allowed clear identification of interaction effects between algae type and inclusion level. In addition, the study extends its relevance by linking experimental findings with regional CH_4_ emission projections.

The results are based on *in vitro* conditions, which may not fully reflect *in vivo* rumen dynamics. Variability related to animal physiology, feed intake behavior, and long-term microbial adaptation was not addressed. The study did not evaluate production parameters such as growth performance or product quality. In addition, variations in phytochemical composition due to environmental and seasonal factors were not explored.

Future research should focus on *in vivo* validation to confirm the effects on CH_4_ mitigation, productivity, and animal health. Long-term studies are needed to assess microbial adaptation and feeding responses. Optimization of processing methods to improve nutrient availability and reduce potential anti-nutritional effects is also required. Economic feasibility and large-scale applicability should be evaluated. Investigating synergistic effects with other feed additives may further enhance fermentation efficiency and CH_4_ reduction.

*S. cristaefolium*, *S. crassifolium*, and *E. acoroides* show strong potential as alternative feed resources capable of improving fermentation efficiency and reducing CH_4_ emissions. Their utilization supports sustainable livestock production and aligns with climate mitigation strategies, particularly in marine-rich regions. With proper validation and implementation, these algae can contribute to environmentally sustainable and economically viable ruminant feeding systems.

## DATA AVAILABILITY

The data generated during the study are included in the manuscript.

## AUTHORS’ CONTRIBUTIONS

NG: Supervision, experiment execution, manuscript drafting, and final manuscript revision. RH and RP: Manuscript drafting, data interpretation, and final manuscript revision. AN: Performed the laboratory analyses and drafted the manuscript. MAP and GAWS: Conducted the statistical analyses and revised the manuscript. All authors have read and approved the final manuscript.
